# *SEMA3A*, a Gene Involved in Axonal Pathfinding, Is Mutated in Patients with Kallmann Syndrome

**DOI:** 10.1371/journal.pgen.1002896

**Published:** 2012-08-23

**Authors:** Naresh Kumar Hanchate, Paolo Giacobini, Pierre Lhuillier, Jyoti Parkash, Cécile Espy, Corinne Fouveaut, Chrystel Leroy, Stéphanie Baron, Céline Campagne, Charlotte Vanacker, Francis Collier, Corinne Cruaud, Vincent Meyer, Alfons García-Piñero, Didier Dewailly, Christine Cortet-Rudelli, Ksenija Gersak, Chantal Metz, Gérard Chabrier, Michel Pugeat, Jacques Young, Jean-Pierre Hardelin, Vincent Prevot, Catherine Dodé

**Affiliations:** 1 Inserm U837, Développement et Plasticité du Cerveau Postnatal, Centre de Recherche Jean-Pierre Aubert, Lille, France; 2 Université Lille Nord de France, Lille, France; 3 UDSL, Ecole de Médecine, Lille, France; 4 Institut Cochin, Département de Génétique et Développement, Inserm U1016, Université Paris-Descartes, Paris, France; 5 Laboratoire de Biochimie et Génétique Moléculaire, Hôpital Cochin, APHP, Paris, France; 6 CHRU Lille, Service de Gynécologie Endocrinienne et Médecine de la Reproduction, Hôpital Roger Salengro, Lille, France; 7 Institut de Génomique, Genoscope, CEA, DSV, Evry, France; 8 Unitat de Rinologia, Hospital Clinic, Barcelona, Spain; 9 CHRU Lille, Service d'Endocrinologie, CHR Lille, Lille, France; 10 Department of Obstetrics and Gynecology, University Medical Center Ljubljana, Ljubljana, Slovenia; 11 Pôle Femme, Mère, et Enfant, CHU Morvan, Brest, France; 12 Service d'Endocrinologie, Hôpital Civil, Strasbourg, France; 13 Service d'Endocrinologie, Hôpital Neurologique et Neurochirurgical, Bron, France; 14 Service d'Endocrinologie, Hôpital Bicêtre, Le Kremlin-Bicêtre, France; 15 Inserm U587, Département de Neuroscience, Institut Pasteur, Université Pierre et Marie Curie – Paris 06, Paris, France; Max Planck Institute for Molecular Genetics, Germany

## Abstract

Kallmann syndrome (KS) associates congenital hypogonadism due to gonadotropin-releasing hormone (GnRH) deficiency and anosmia. The genetics of KS involves various modes of transmission, including oligogenic inheritance. Here, we report that *Nrp1* ^sema/sema^ mutant mice that lack a functional semaphorin-binding domain in neuropilin-1, an obligatory coreceptor of semaphorin-3A, have a KS–like phenotype. Pathohistological analysis of these mice indeed showed abnormal development of the peripheral olfactory system and defective embryonic migration of the neuroendocrine GnRH cells to the basal forebrain, which results in increased mortality of newborn mice and reduced fertility in adults. We thus screened 386 KS patients for the presence of mutations in *SEMA3A* (by Sanger sequencing of all 17 coding exons and flanking splice sites) and identified nonsynonymous mutations in 24 patients, specifically, a frameshifting small deletion (D538fsX31) and seven different missense mutations (R66W, N153S, I400V, V435I, T688A, R730Q, R733H). All the mutations were found in heterozygous state. Seven mutations resulted in impaired secretion of semaphorin-3A by transfected COS-7 cells (D538fsX31, R66W, V435I) or reduced signaling activity of the secreted protein in the GN11 cell line derived from embryonic GnRH cells (N153S, I400V, T688A, R733H), which strongly suggests that these mutations have a pathogenic effect. Notably, mutations in other KS genes had already been identified, in heterozygous state, in five of these patients. Our findings indicate that semaphorin-3A signaling insufficiency contributes to the pathogenesis of KS and further substantiate the oligogenic pattern of inheritance in this developmental disorder.

## Author Summary

Kallmann syndrome is a hereditary developmental disease that affects both the hormonal reproductive axis and the sense of smell. There is a developmental link between the reproductive and olfactory disorders: neuroendocrine cells producing the gonadotropin-releasing hormone that is deficient in the patients normally migrate from the nose to the forebrain along olfactory nerve fibers during embryonic life, and they fail to do so in the patients. Affected individuals usually do not undergo spontaneous puberty. Hormone replacement therapy is the treatment to initiate virilization in males or breast development in females and later to develop fertility in both sexes. This is a genetically heterogeneous disease. Mutations in any of eight causative genes identified so far have been found in approximately 30% of the affected individuals, thus indicating that other genes remain to be discovered. We report on the identification, in 6% of the KS patients, of various loss-of-function mutations in the gene coding for semaphorin-3A, a secreted protein involved in the navigation of olfactory nerve fibers during embryogenesis. The fact that many of these mutations were also detected in clinically unaffected individuals indicates that they must combine with other genetic defects to produce the disease phenotype.

## Introduction

Kallmann syndrome (KS, MIM 147950, 244200, 308700, 610628, 612370, 612702) is an inherited neurodevelopmental disorder defined as the association of hypogonadotropic hypogonadism, due to gonadotropin-releasing hormone (GnRH) deficiency, and the inability to smell (anosmia or hyposmia), related to abnormal development of the peripheral olfactory system (olfactory nerves and olfactory bulbs). The genetics of KS involves various modes of transmission, specifically, autosomal recessive, autosomal dominant with incomplete penetrance, X-chromosome linked, and also oligogenic inheritance [1], [2]. Pathohistological studies of fetuses with olfactory bulb agenesis have shown that the reproductive phenotype of KS results from a pathological sequence in embryonic life, whereby premature interruption of the olfactory, vomeronasal and terminal nerve fibers in the frontonasal region disrupts the migration of neuroendocrine GnRH cells, which normally migrate from the nose to the brain along these nerve fibers [3], [4]. What causes the primary failure of these fibers to establish proper contact with the forebrain is, however, still unknown. Since KS is genetically heterogeneous, identification of the various genes involved and the study of appropriate animal models are expected to provide valuable clues. Barely 30% of the KS patients have mutations in any of the eight genes known so far, specifically, *KAL1* (ID 3730) [5]–[7], *FGFR1* (ID 2260) [8], *FGF8* (ID 2253) [9], *PROKR2* (ID 128674), *PROK2* (ID 60675) [10], *WDR11* (ID 55717) [11], *HS6ST1* (ID 9394) [12], *CHD7* (ID 55636) [13], [14], and current efforts thus concentrate on the identification of other genes that contribute to this disorder. One strategy is based on close pathohistological examination of targeted mutant mice that may reproduce the human KS phenotype. Here, we show that *Nrp1* ^sema/sema^ mutant mice, which are defective for the semaphorin-binding domain of the membrane coreceptor neuropilin-1, have a KS-like phenotype, and we provide genetic evidence that insufficient semaphorin-3A signaling can contribute to the KS phenotype in man.

## Results/Discussion

### Neuropilin-1 expression delineates the migratory route of embryonic GnRH cells in mice and humans

**Figure 1:**
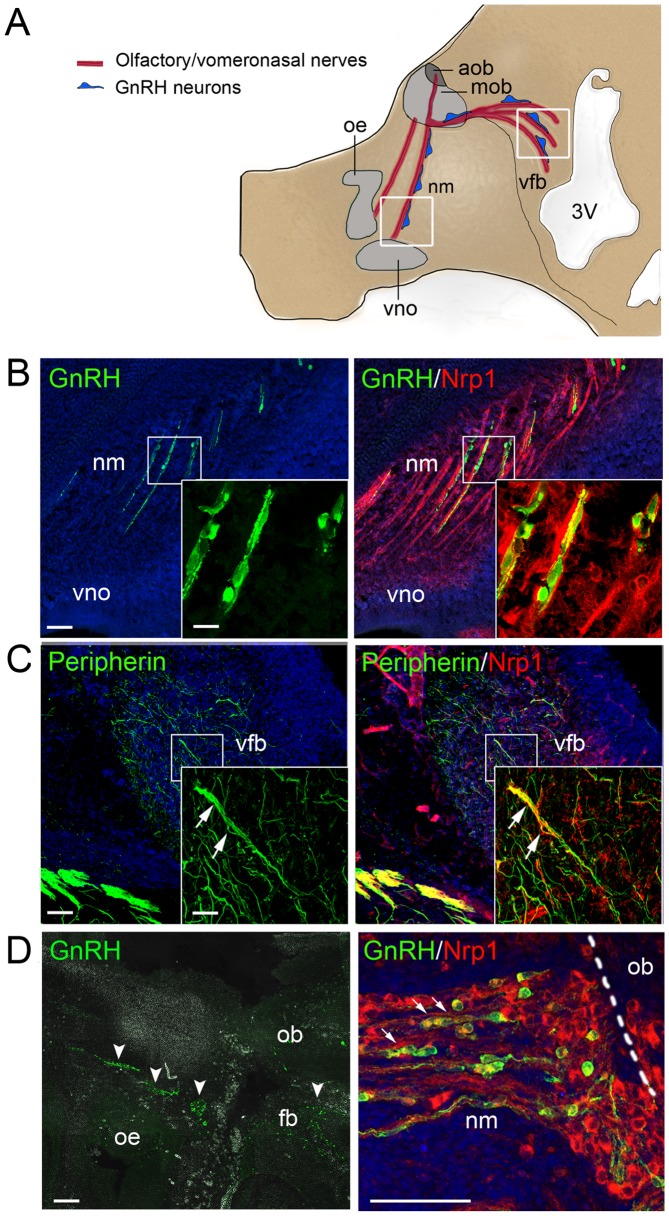
Expression of the Sema3A coreceptor Nrp1 by vomeronasal/terminal nerve fibers and migrating GnRH cells in human and mouse embryos. (A) Schematic representation of the head of a mouse embryo at E14.5, showing the scaffold of vomeronasal/terminal nerve fibers (in red) along which GnRH cells (in blue) migrate from the nose to the ventral forebrain region. Several areas along this migratory path have been shown to produce Sema3A, including the frontonasal mesenchyme and the olfactory bulb region [21], [33]. Boxes indicate the locations of the sagittal sections shown in (B) and (C). Abbreviations: oe, olfactory epithelium; vno, vomeronasal organ; nm, frontonasal mesenchyme; mob, main olfactory bulb; aob, accessory olfactory bulb; vfb, ventral forebrain; 3V, third ventricle. (B) Sagittal section of the frontonasal region in an E14.5 mouse embryo. In the frontonasal mesenchyme (nm), migrating GnRH-immunoreactive cells (green) are morphologically associated with Nrp1-immunoreactive nerve fibers (red) originating in the vomeronasal organ (vno). Single plane confocal images at higher magnification (insets) show that GnRH cells are Nrp1-immunoreactive (green+red = yellow staining). (C) Sagittal section of the ventral forebrain (vfb) in an E14.5 mouse embryo. The peripherin-immunoreactive (green) fibers of the caudal branch of the vomeronasal nerve (arrows) are also Nrp1-immunoreactive (red), as shown by their yellow staining (green+red). (D) Sagittal section of the olfactory epithelium (oe) and olfactory bulb (ob) regions (left panel) and detail of the frontonasal region (right panel) in a 9 week-old human fetus. Clusters of GnRH-immunoreactive cells (green, arrowheads) are visible in the frontonasal mesenchyme (nm) and the rostral forebrain (fb). In the frontonasal region, these cells migrate in close contact with Nrp1-immunoreactive axons (red). Note that migrating GnRH cells are also Nrp1-immunoreactive, as shown by their yellow staining (green+red) in the right panel (arrows). Scale bars: 100 µm (25 µm in insets).

In the mouse, GnRH cells begin to leave the epithelium of the medial olfactory pit around embryonic day 11.5 (E11.5). They migrate in the frontonasal region in close association with growing fibers of the vomeronasal and terminal nerves, then penetrate into the rostral forebrain together with the central processes of these nerves, and continue their migration towards the hypothalamic region along a branch of the vomeronasal nerve that projects to the basal forebrain or along fibers of the terminal nerve itself [15]–[17] (Figure 1A). Proper navigation of growing axons depends on guidance cues, which include semaphorins, a large and diverse family of secreted and membrane-associated proteins [18]. Among these, there is semaphorin-3A (Sema3A), a secreted protein with repulsive effects on primary olfactory axons expressing the coreceptor neuropilin-1 (Nrp1) [19]–[21]. The role of semaphorins in the navigation of vomeronasal/terminal axons and embryonic GnRH cells is still unclear, but previous studies in rodents have shown that migrating GnRH cells are morphologically associated with Nrp1-immunoreactive axons and are themselves immunoreactive [22], [23]. Indeed, we were able to confirm these findings in E14.5 mouse embryos, and extend them to a 9-week old human fetus (Figure 1B–1D), using specific antibodies to Nrp1 (Figure S1) in immunohistofluorescence experiments. Notably, the caudal branch of the vomeronasal nerve that accompanies GnRH cells in their intracerebral path was also Nrp1-immunoreactive in the mouse embryos (Figure 1C). These observations suggested that semaphorin signaling through Nrp1 imparts guidance information to axons of the vomeronasal neurons and migrating GnRH cells.

### Migration of GnRH cells to the basal forebrain is defective in *Nrp1* ^sema/sema^ mutant mice

**Figure 2:**
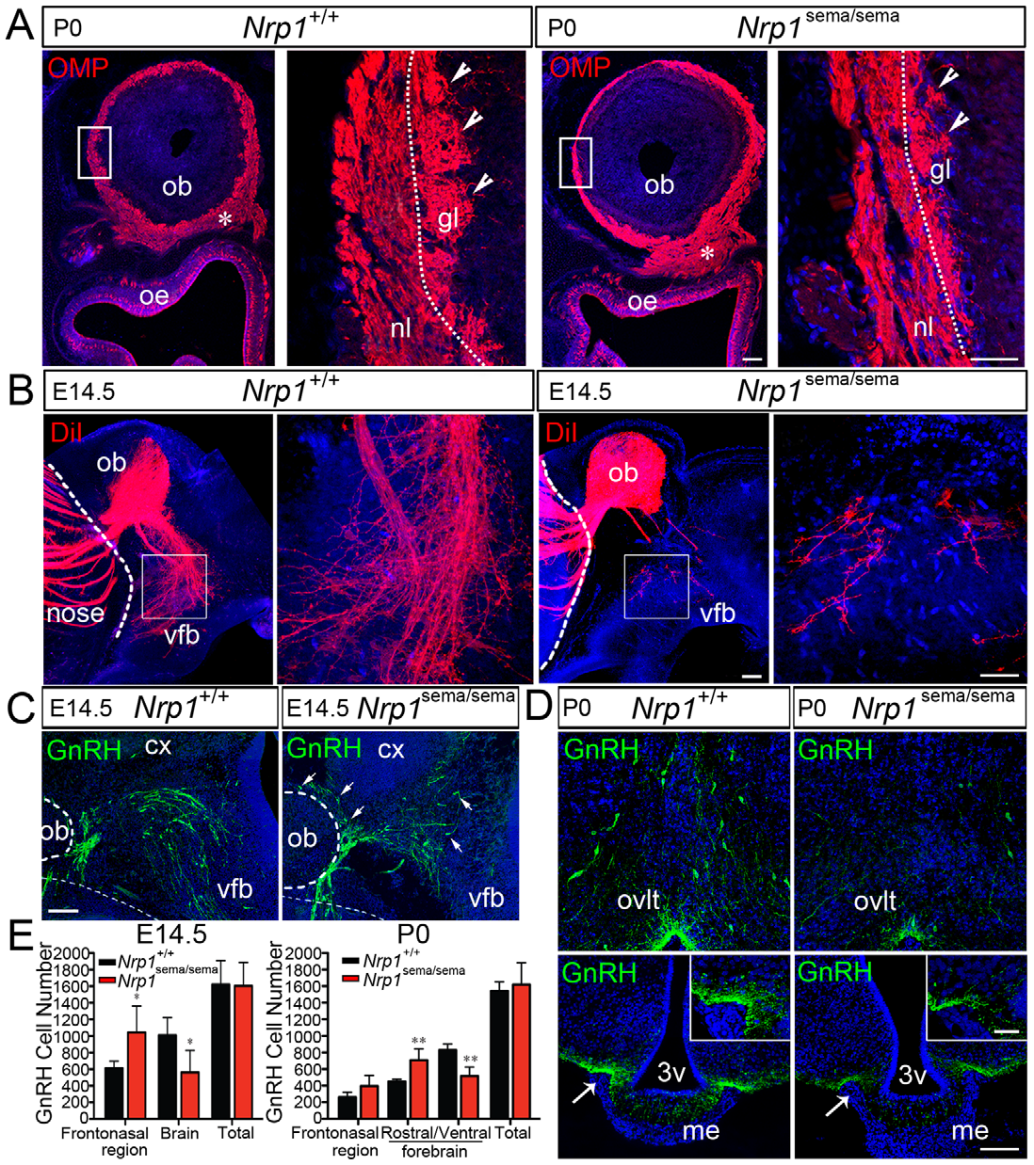
Defects in olfactory and vomeronasal axons, and GnRH cell migration in *Nrp1* ^sema/sema^ mutant mice. (A) Coronal sections of the right olfactory epithelium (oe) and olfactory bulb (ob) regions (left panels), and detail of the olfactory bulb showing the olfactory nerve layer (nl) and glomerular layer (gl) (right panels) in *Nrp1* ^+/+^ and *Nrp1* ^sema/sema^ newborn (P0) mice. Axons of the olfactory receptor neurons were immunostained (red) using an antibody directed against the olfactory marker protein (OMP). In the *Nrp1* ^sema/sema^ mouse, the immunostaining is both enlarged below the olfactory bulb ventro-medial aspect (asterisks) and markedly reduced in the glomerular layer (arrowheads) compared to wild-type. (B) Sagittal sections of the rostral and ventral forebrain regions (left panels), and detail of the caudal branch of the vomeronasal nerve (right panels) in *Nrp1* ^+/+^ and *Nrp1* ^sema/sema^ E14.5 mouse embryos. A crystal of the DiI lipophilic fluorescent dye has been placed in the vomeronasal organ lumen to anterogradely label vomeronasal axons. The vomeronasal nerve extends across the medial aspect of the olfactory bulb and projects both dorsally, to the accessory olfactory bulb, and caudally, to the ventral forebrain (vfb). In the mutant mouse, fibers in the caudal branch are scarce compared to wild-type. (C) Sagittal sections of the rostral and ventral forebrain regions at E14.5, immunostained for GnRH (green). Note the abnormal distribution of GnRH-immunoreactive cells in the *Nrp1* ^sema/sema^ mouse (arrows). (D) Coronal sections of the preoptic region (upper panels) showing GnRH neuroendocrine cells (green) and their projections in the median eminence (me, arrows) (lower panels) in *Nrp1* ^+/+^ and *Nrp1* ^sema/sema^ newborn (P0) mice. The immunostaining is reduced in the *Nrp1* ^sema/sema^ mouse. (E) Quantitative analysis (mean ± s.d.) of GnRH cell distributions in *Nrp1* ^+/+^ and *Nrp1* ^sema/sema^ mice at E14.5 and P0. * and ** denote statistically significant differences between genotypes in the indicated head regions (two-way ANOVA followed by Tukey's range test) with *p*<0.05 and *p*<0.01, respectively. Note that the total numbers of GnRH cells are not statistically different between *Nrp1* ^+/+^ and *Nrp1* ^sema/sema^ mice at E14.5 or P0 (Student's t-test, *p*>0.05). Other abbreviations: cx, cerebral cortex; ovlt, organum vasculosum of lamina terminalis; 3v, third ventricle. Scale bars: 100 µm (50 µm in inset).

We thus analyzed *Nrp1* ^sema/sema^ mutant mice that harbor inactivating aminoacid substitutions in the semaphorin-binding domain of Nrp1. Unlike *Nrp1* ^−/−^ knockout mice, which die around E12.5 [24], these mice survive until birth [25]. In *Nrp1* ^sema/sema^ newborn mice (n = 4), many axons of olfactory receptor neurons were stuck at the dorsal aspect of the cribriform plate and did not project to the olfactory bulb glomeruli (Figure 2A). Olfactory cues are thought to play an important role in suckling behavior [26]. Analysis of six litters at postnatal day 1 (P1) indeed showed that 7 out of 8 *Nrp1* ^sema/sema^ pups had little or no milk in their stomachs, whereas most *Nrp1^+^* ^/+^ and *Nrp1* ^sema/+^ littermates (18 out of 21) had full stomachs. These findings account for the decreased survival rate of homozygous, but not heterozygous, mutant pups [25], and strongly suggest that the sense of smell is affected in *Nrp1* ^sema/sema^ mice. Most importantly, DiI axonal labeling at E14.5 showed abnormal projection of the vomeronasal nerve to the ventral forebrain in the homozygous mutant embryos (n = 4) (Figure 2B). Since this projection forms the axonal scaffold for the intracerebral migration of GnRH cells [17], [27], we analyzed the distribution of these cells in E14.5 and newborn mice. At E14.5, a significant accumulation of GnRH cells in the nasal compartment and concomitant decreased cell number within the brain already indicated abnormal cell migration in the mutants (n = 4) (Figure 2E). In addition, while GnRH cells normally turn ventrally towards the basal forebrain, in *Nrp1* ^sema/sema^ embryos, many GnRH cells were found to migrate dorsally and medially towards the cortex and the thalamus, respectively, along aberrantly projecting axonal fibers (Figure 2C, Figure S2). Incidentally, conditional mutant mice that lack Nrp1 only in GnRH cells (*GnRH::cre*; *Nrp1* ^loxP/loxP^ mice) displayed a normal distribution of these cells between the nose and the brain at E14.5 as well as a normal number of these cells in the adult brain (Figure S3 and data not shown), thus confirming that the defective migration we found in *Nrp1* ^sema/sema^ embryos is not a cell-autonomous trait. The migration defect was still conspicuous at birth (Figure 2D), a time when neuroendocrine GnRH cells have completed their migration in normal mice [3]. The ventral forebrain region of *Nrp1* ^sema/sema^ newborn mice (n = 4) indeed contained 38% fewer GnRH cells, which were dispersed, while there was a 36% increase in the number of GnRH cells detected in the rostral forebrain compared with *Nrp1^+^* ^/+^ littermates (n = 5, *p*<0.01 for both comparisons) (Figure 2E). This GnRH-cell migration defect in *Nrp1* ^sema/sema^ animals resulted in decreased GnRH immunoreactivity in the median eminence of the hypothalamus (Figure 2D), which is the projection field of neuroendocrine GnRH cells.

Of the *Nrp1* ^sema/sema^ newborn mice, only four males and two females survived into adulthood. Both females had delayed pubertal activation, specifically, the first ovulation occurred more than 10 days later than in *Nrp1* ^sema/+^ heterozygous littermates, and monitoring of the ovarian cycle from P60 showed that one female stayed in the diestrous stage (a stage with low gonadotropin outputs) throughout the 3-week study period, while the other female had disrupted ovarian cyclicity (data not shown). Male reproductive capacity was assessed by breeding the young adult (P90) *Nrp1* ^sema/sema^ males with confirmed wild-type dams, and monitoring the occurrence of litters over 10–13 months. While *Nrp1* ^sema/+^ males (n = 4) produced about one litter per month, as did *Nrp1^+^* ^/+^ males, the fertility index (number of litters per month) was markedly reduced in the *Nrp1* ^sema/sema^ males, which only gave birth to 2 to 4 litters (fertility index: 0.29±0.04 *vs.* 1.08±0.12 in *Nrp1^sema^* ^/+^; Student's t-test, *p*<0.001). Moreover, neuroanatomical analysis of *Nrp1* ^sema/sema^ adult brains showed significantly reduced GnRH cell populations in the preoptic and hypothalamic regions (384±67 GnRH cells, n = 4) compared to *Nrp1* ^sema/+^ littermates (767±49 GnRH cells, n = 4; Student's t-test, *p*<0.001), whereas *Nrp1* ^sema/+^ mice did not differ from *Nrp1* ^+/+^ mice (701±11 GnRH cells, n = 4; Student's t-test, *p*>0.05). Therefore, the GnRH cell migration defect found in *Nrp1* ^sema/sema^ mouse embryos was not corrected during later development, and caused subfertility in adult homozygous mutants.

### *SEMA3A* loss-of-function mutations in Kallmann syndrome patients

**Figure 3:**
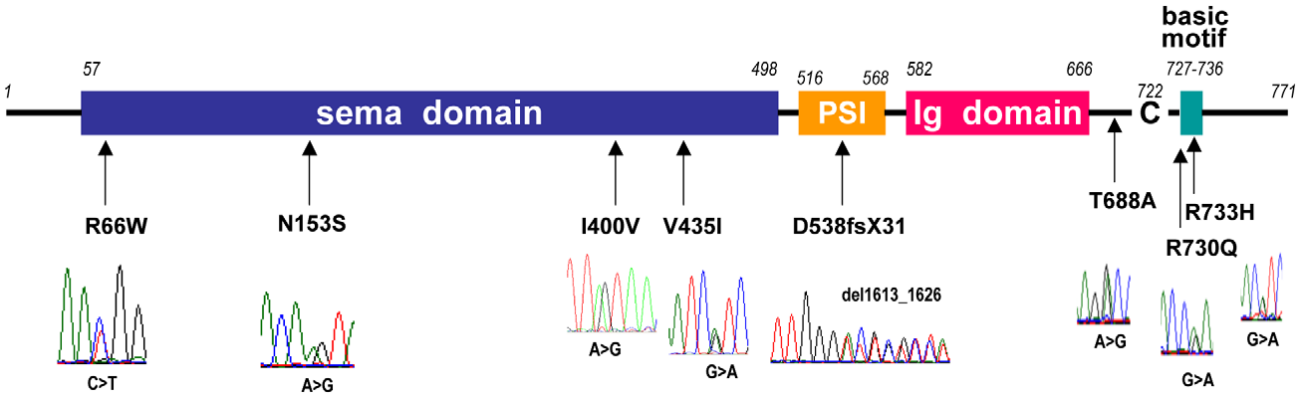
Diagram of Sema3A with the mutations found in Kallmann syndrome patients. Sequence chromatograms of the mutations are shown together with the positions of the corresponding aminoacid residues in the protein domains. Abbreviations: sema, semaphorin; PSI, plexin/semaphorin/integrin; Ig, immunoglobulin-like; C, cysteine residue involved in Sema3A dimerization (interchain disulfide bond) [18].

**Figure 4:**
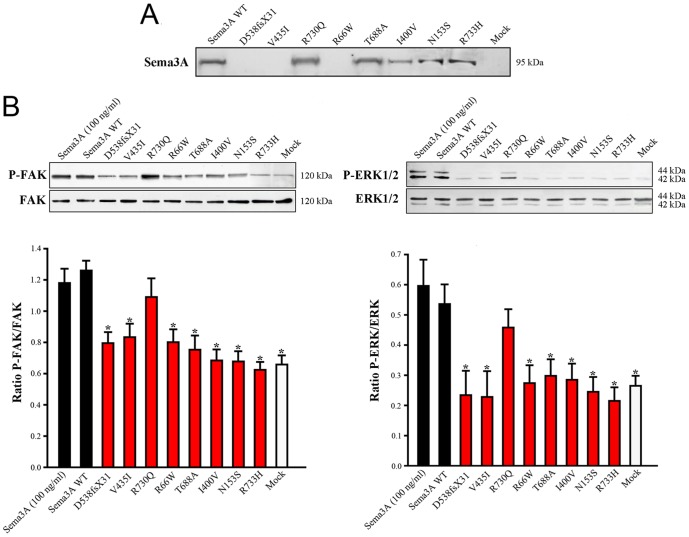
Defective secretion or signaling activity of Sema3A proteins harboring the mutations identified in Kallmann syndrome patients. (A) Western blot analysis of conditioned media from transfected COS-7 cells producing wild-type (WT) or mutated Sema3A proteins. The p.D538fsX31 frameshifting mutation, and the p.V435I and p.R66W missense mutations result in the absence of a secreted protein. (B) Upper panels: Representative western blots for the phosphorylated and total forms of FAK (left panel) and ERK1/2 (right panel) in GN11 cells following a 20 min incubation with serum-free medium (mock, negative control), 100 ng/ml of purified recombinant human Sema3A, or the conditioned media from transfected COS-7 cells producing wild-type or mutated Sema3A proteins. Lower panels: Bar graphs illustrate the mean ratio (± s.d.) of the western blot signal intensity obtained for phosphorylated FAK (P-FAK) or ERK1/2 (P-ERK1/2) to that of total FAK or ERK1/2, respectively. Each experiment was carried out three times independently. * denotes statistically significant difference with wild-type Sema3A (one-way ANOVA followed by Fisher's LSD test, *p*<0.05). a.u.: arbitrary units (pixel density).

The KS-like phenotype of *Nrp1* ^sema/sema^ mice, and that, even more pronounced, of *Sema3a* ^−/−^ mice [22], prompted us to ask whether insufficient Sema3A signaling through Nrp1 might also be involved in the human disorder. We sought mutations, by Sanger sequencing, in the 17 coding exons of *SEMA3A* (ID 10371) and flanking splice sites, in 386 unrelated KS patients (297 males and 89 females). All of them had confirmed hypogonadotropic hypogonadism and anosmia or hyposmia, and some already harbored a mutation in one of the five KS genes we had previously analyzed, specifically, in *KAL1* (13 patients), *FGFR1* (30 patients), *FGF8* (3 patients), *PROKR2* (30 patients), or *PROK2* (12 patients). Nonsynonymous mutations in *SEMA3A* were found in 24 patients (20 males and 4 females), all in heterozygous state (Table 1). They consist of a frameshifting deletion of 14 nucleotides (c.del1613_1626; p.D538fsX31), and seven different missense mutations (p.R66W, p.N153S, p.I400V, p.V435I, p.T688A, p.R730Q, p.R733H) that affect evolutionarily conserved aminoacid residues located in different domains of the protein (Figure 3). In addition, the p.R730Q and p.R733H mutations, which both remove basic residues in the C-terminal basic motif of Sema3A, are predicted to affect in vivo proteolytic processing by furin-like endoproteases at residue R734 [28]. Notably, all the missense mutations, but not the frameshifting mutation, have been reported in the Exome Variant Server database, with allele frequencies in the European American population below 0.03% except for p.N153S (0.4%) and p.V435I (1.3%). Three of these mutations (p.R66W, p.V435I, p.R730Q) were also detected in our sample of 386 unrelated Caucasian controls (see Table 1). We thus studied the effects of the eight mutations on the signaling activity of Sema3A using the GN11 cell line, derived from murine embryonic GnRH cells [29]–[31], and conditioned media from transfected COS-7 cells producing Sema3A either from the wild-type *SEMA3A* cDNA or from cDNAs harboring the mutations. We found that the conditioned medium from COS-7 cells transfected with the wild-type *SEMA3A* cDNA was as potent at inducing phosphorylation of FAK (focal adhesion kinase) and ERK1/2 (extracellular signal-regulated kinases 1 and 2) in GN11 cells as the purified recombinant human Sema3A (100 µg/L). By contrast, Sema3As harboring the N153S, I400V, T688A, or R733H missense mutations were ineffective, despite normal production and secretion of the proteins by COS-7 cells, shown by western blot analysis of the conditioned media. The R66W and V435I mutant proteins were not detected in the conditioned medium, which indicates defective secretion. Likewise, the c.del1613_1626 (p.D538fsX31) frameshifting mutation resulted in the absence of protein secretion, as expected (Figure 4). From these results, we were able to conclude that all the mutations, except p.R730Q, are loss-of-function mutations that affect the secretion or signaling activity of Sema3A, which strongly argues in favor of their pathogenic effect in the KS patients. In addition, the p.R730Q mutation may still have a pathogenic effect not detected in our experimental system, especially since this mutation is expected to impair proteolytic processing of Sema3A in vivo, as mentioned previously. Notably, the patients carrying the p.T688A and p.I400V mutations, and three patients carrying the p.V435I mutation also carry, in heterozygous state, p.Y217D, p.R268C (two patients), p.H70fsX5, and p.G687N pathogenic mutations in *KAL1*, *PROKR2*, *PROK2*, and *FGFR1*, respectively (Table 1), which further substantiates the digenic/oligogenic mode of inheritance of KS [1], [2]. Based on the seemingly normal reproductive phenotype of *Sema3a* ^+/−^ heterozygous mice [21], [22], we suggest that the monoallelic mutations in *SEMA3A* are not sufficient to induce the abnormal phenotype in the patients, but contribute to the pathogenesis of KS through synergistic effects with mutant alleles of other disease-associated genes. Accordingly, the other KS patients who carry monoallelic mutations in *SEMA3A* are also expected to carry at least one pathogenic mutation in another gene (see footnote). Although *NRP1* (ID 8829) might be viewed as one of the best candidates, we did not find a mutation within its 17 coding exons and flanking splice sites in any of these patients, nor did we in a group of 100 KS patients without *SEMA3A* mutations, which indicates that mutations in *NRP1*, if any, are infrequent. It is also possible that some of the additional mutations affect other proteins involved in Sema3A-signaling, such as members of the plexin family of transmembrane receptors or neuropilin-2 [18], [22]. A whole-exome sequencing strategy should prove useful to explore the spectrum of genes which, when mutated, can lead to a KS phenotype in conjunction with *SEMA3A* mutations.

**Table 1:** *SEMA3A* mutations identified in Kallmann syndrome patients.

Nucleotide change	Exon	Aminoacid change	Protein domain	Allele frequency in control subjects	Gender (M/F) of patients	Additional mutation in the patient
c.197C>T	2	p.R66W	sema	1/772	M	
c.458A>G	5	p.N153S	“	0/772	2M	
c.1198A>G	11	p.I400V	“	0/772	M	*PROKR2* p.R268C
c.1303G>A	“	p.V435I	“	13/772	11M+2F	
“	“	“	“	“	M	*PROKR2* p.R268C
“	“	“	“	“	F	*PROK2* p.H70fsX5
“	“	“	“	“	M	*FGFR1* p.G687R
c.del1613_1626	14	p.D538fsX31	PSI	0/772	M	
c.2062A>G	17	p.T688A	interdomain	0/772	M	*KAL1* p.Y217D
c.2189G>A	“	p.R730Q	basic motif	1/772	F	
c.2198G>A	“	p.R733H	“	0/772	M	

### Note

While this article was under review, Young et al. reported the coexistence of KS and a large deletion in *SEMA3A*, in heterozygous state, in two siblings and their clinically affected father (*Hum. Reprod.*, 2012; 27:1460–1465). Our findings do not support mere autosomal dominant Mendelian inheritance in this family, and suggest that another, as yet unidentified genetic hit combines with *SEMA3A* haploinsufficiency to produce the disease phenotype.

## Materials and Methods

### Ethics statement

This study was approved by the national research ethics committee (agence de biomédecine, Paris, France).

### Animals and human fetus

All experiments on mice were carried out in accordance with Directive 86/609/EEC of the Council of the European Communities regarding the mammalian research and French bylaw. *Nrp1* ^sema/+^ mice (B6.129(C)-Nrp1tm1Ddg/J) [25] were purchased from the Jackson laboratory (Maine, USA), maintained on a controlled 12 h∶12 h light cycle, provided with food and water ad libitum, and genotyped as described previously [25]. E14.5 (plug day, E0.5), P0, and adult *Nrp1* ^+/+^, *Nrp1* ^sema/+^ and *Nrp1* ^sema/sema^ mice were obtained and processed for immunohistofluorescence analyses as previously described [30]. In addition, homozygous *Nrp1* ^loxP/loxP^ mice (B6.129(SJL)-Nrp1tm2Ddg/J) [25] from the Jackson laboratory were crossed with a transgenic mouse line expressing the cre recombinase under the control of the GnRH gene promoter (*GnRH::cre* mice) [32], a gift from C. Dulac (Harvard university, Cambridge, USA), to obtain *GnRH::cre*; *Nrp1* ^loxP/loxP^ mice that lack Nrp1 in GnRH cells only. *Nrp1* ^loxP/loxP^ and *GnRH::cre*; *Nrp1* ^loxP/loxP^ mice were used for immunohistofluorescence analyses at E14.5 and adult stages.

The human fetus was obtained from a voluntary terminated pregnancy, with parent's written informed consent. Gestational age was established by crown-rump length measurement. The fetus was fixed in 4% paraformaldehyde in 0.1 M phosphate buffered saline (PBS), pH 7.4, for three weeks at 4°C, and then immersed in 0.1 M PBS containing 30% sucrose for two days at 4°C. The head was embedded in OCT embedding medium (Tissue-Tek), frozen, and sagittal cryosections (20 µm thick) were cut and processed for immunohistofluorescence.

### Immunohistofluorescence

Immunohistofluorescence experiments were carried out as described previously [30]. Primary antibodies were: rabbit anti-GnRH (dilution 1∶3000), a gift from G. Tramu (University of Bordeaux, France); rabbit anti-peripherin (dilution 1∶1000), AB1530 (Millipore); goat anti-neuropilin1 (dilution 1∶400), AF566 (R & D systems); goat anti-olfactory marker protein (dilution 1∶6000), a gift from F. L. Margolis (University of Maryland, Baltimore, USA).

### DiI labeling of nerve fibers

Vomeronasal nerve fibers were traced anterogradely with the lipophilic fluorescent dye DiI (1,1′-dioctadecyl-3,3,3′,3′-tetramethylindocarbocyanine perchlorate, Molecular Probes) as previously described [17]. After diffusion of the tracer, serial sagittal sections (100 µm thick) were cut through the forebrain, and analyzed using a LSM 710 confocal microscope (Zeiss) and the ImageJ analysis software (NIH, Bethesda, USA).

### Cell cultures

COS-7 cells and GN11 cells were grown in monolayers in 5% CO_2_ at 37°C, in Dulbecco's modified Eagle's medium (Life Technologies, Inc.) containing 1 mM sodium pyruvate, 2 mM glutamine, 50 mM glucose, and supplemented with 10% fetal bovine serum (Invitrogen), 100 µg/ml streptomycin and 100 U/ml penicillin.

### Signaling activity of wild-type and mutant Sema3A in GN11 cells

A cDNA containing the entire coding region of the human *SEMA3A* (GenBank NM_006080) was inserted into a pRK5 plasmid expression vector. Recombinant plasmids containing *SEMA3A* cDNAs harboring each of the eight mutations identified in the KS patients were then engineered using the QuickChange mutagenesis protocol (Stratagene). COS-7 cells were transiently transfected using a fast-forward protocol (Lipofectamine 2000, Invitrogen) [30]. Conditioned medium was collected 48 h after transfection, tested for the presence of Sema3A by western blot analysis using an anti-Sema3A antibody (Santa Cruz, sc-10720, dilution 1∶100), and then processed for signaling activity experiments in the GN11 cell line. Briefly, subconfluent GN11 cells were grown overnight in serum-free medium, and then stimulated for 20 min with human recombinant Sema3A (R&D systems) at 100 µg/L, or with the concentrated conditioned media from transfected COS-7 cells. Western blot experiments [30] were carried out on cell lysates using antibodies to P-ERK (#9101L) and ERK (#9102L) from Cell Signaling (dilution 1∶1000), or P-FAK (sc56901) and FAK (sc81493) from Santa Cruz (dilution 1∶500).

### DNA sequencing

Informed consent was obtained from all individuals analyzed. Genomic DNAs were prepared from white blood cells using a standard procedure. Each of the *SEMA3A* and *NRP1* coding exons and flanking splice sites was PCR-amplified from genomic DNA using a specific primer pair (see Tables S1 and S2 for primer sequences), and sequenced using either PCR oligonucleotide as sequencing primer. The mutations were confirmed by sequencing two independent PCR products on both DNA strands. Exons 2, 5, 11, 14, and 17 of *SEMA3A*, which harbor the mutations identified in some patients, were analyzed by denaturing high performance liquid chromatography (DHPLC) scanning on an automated HPLC instrument (Wave technology) in 386 unrelated Caucasian controls, followed by Sanger sequencing of the exon in case of abnormal DHPLC profile.

## Supporting Information

Figure S1 The anti-neuropilin1 (Nrp1) polyclonal antibody AF566 (R & D systems) selectively recognizes the semaphorin-binding domain of the protein. Top panel: western blot analysis of Nrp1 in protein extracts from the hypothalamus of *Nrp1* ^+/+^, *Nrp1* ^sema/+^ and *Nrp1* ^sema/sema^ mice (antibody used at 1∶1000 dilution). Bottom panel: immunohistofluorescence analysis of Nrp1 in the median eminence of *Nrp1* ^+/+^ and *Nrp1* ^sema/sema^ newborn mice (antibody used at 1∶400 dilution). Scale bar: 200 µm.
(TIF)

Figure S2 Many GnRH cells migrate along ectopic nerve fibers in the brain of *Nrp1* ^sema/sema^ mutant mice. Immunohistofluorescence analysis of sagittal sections of the rostral and ventral forebrain regions in *Nrp1* ^+/+^ and *Nrp1* ^sema/sema^ mice at E14.5, with anti-GnRH (green) and anti-peripherin (red) antibodies. Insets show detailed views of the normal and the aberrant GnRH cell migratory pathway in the wild-type and the mutant mouse, respectively. In both cases, migrating GnRH cells appear to follow peripherin-immunoreactive axonal fibers (arrows). Abbreviations: cx, cortex; nm, frontonasal mesenchyme; ob, olfactory bulb; vfb, ventral forebrain. Scale bar: 50 µm (20 µm in insets).
(TIF)

Figure S3 GnRH cell migration is not affected in *GnRH::cre*; *Nrp1* ^loxP/loxP^ conditional knockout mice that lack Nrp1 only in GnRH cells. (A) Immunohistofluorescence analysis of the frontonasal region (sagittal sections, single plane confocal microscopy images) in *Nrp1* ^loxP/loxP^ and *GnRH::cre*; *Nrp1* ^loxP/loxP^ mice at E14.5, with anti-GnRH (green) and anti-Nrp1 (red) antibodies. As expected, Nrp1 immunoreactivity of the GnRH cells (yellow) is detected in the *Nrp1* ^loxP/loxP^ mouse, but not in the *GnRH::cre*; *Nrp1* ^loxP/loxP^ mouse. Abbreviations: ob, olfactory bulb; fb, forebrain. Scale bar: 50 µm (20 µm in insets). (B) *Nrp1* ^loxP/loxP^ and *GnRH::cre*; *Nrp1* ^loxP/loxP^ mice display similar distributions of GnRH cells between the nose and the brain at E14.5 (Kruskal-Wallis test, *p*>0.05).
(TIF)

Table S1 *SEMA3A* sequencing primers.
(DOCX)

Table S2 *NRP1* sequencing primers.
(DOCX)
