# Supplementary figures and images for: SEMA3A, a Gene Involved in Axonal Pathfinding, Is Mutated in Patients with Kallmann Syndrome
Source: PLoS Genet. 2012 Aug 23;8(8):e1002896. doi: 10.1371/journal.pgen.1002896 (PMC3426548; doi:10.1371/journal.pgen.1002896)

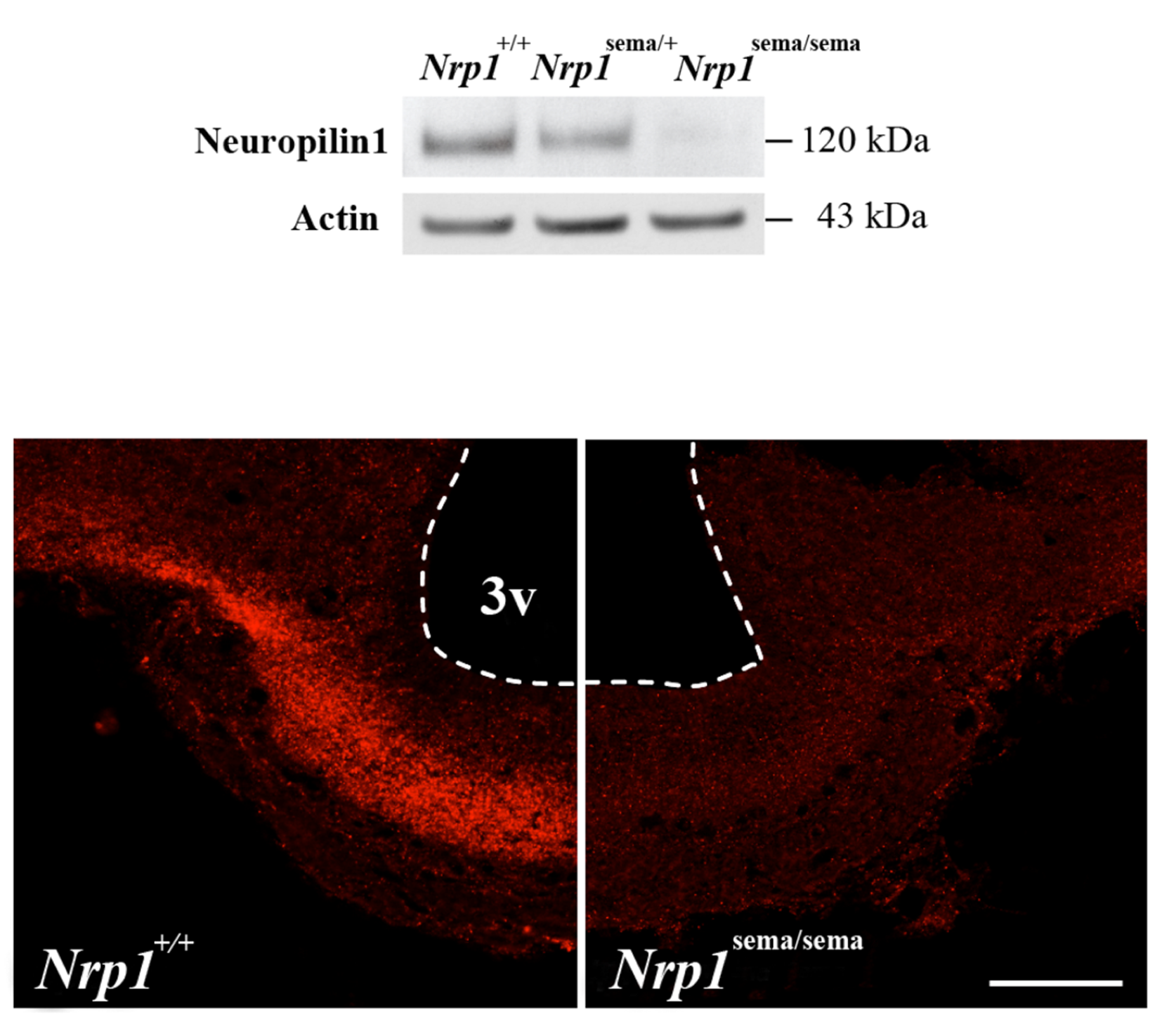

Supplement: Figure S1 — The anti-neuropilin1 (Nrp1) polyclonal antibody AF566 (R & D systems) selectively recognizes the semaphorin-binding domain of the protein. Top panel: western blot analysis of Nrp1 in protein extracts from the hypothalamus of Nrp1 +/+, Nrp1 sema/+ and Nrp1 sema/sema mice (antibody used at 1∶1000 dilution). Bottom panel: immunohistofluorescence analysis of Nrp1 in the median eminence of Nrp1 +/+ and Nrp1 sema/sema newborn mice (antibody used at 1∶400 dilution). Scale bar: 200 µm. (TIF) [file pgen.1002896.s001.tif]

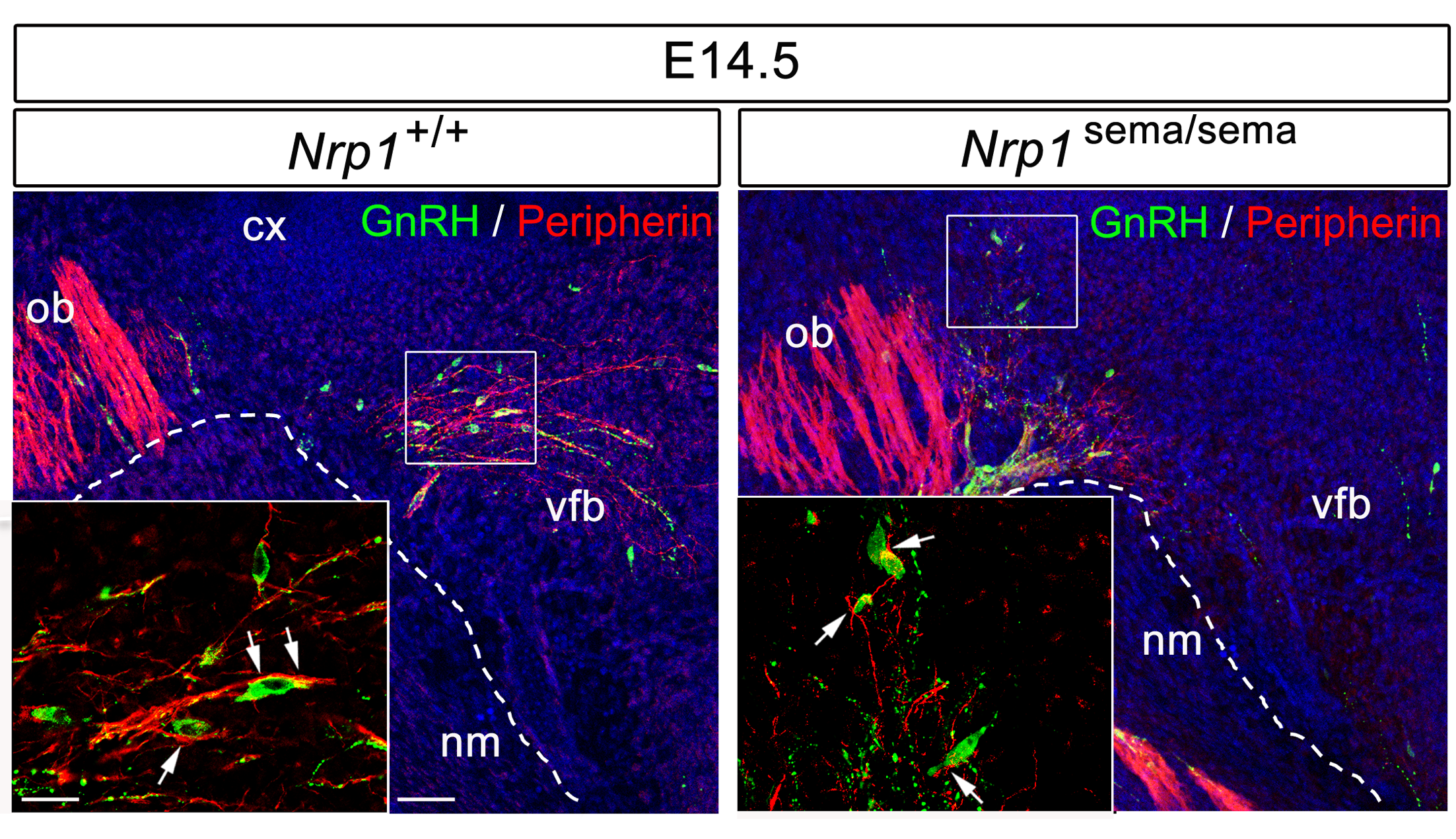

Supplement: Figure S2 — Many GnRH cells migrate along ectopic nerve fibers in the brain of Nrp1 sema/sema mutant mice. Immunohistofluorescence analysis of sagittal sections of the rostral and ventral forebrain regions in Nrp1 +/+ and Nrp1 sema/sema mice at E14.5, with anti-GnRH (green) and anti-peripherin (red) antibodies. Insets show detailed views of the normal and the aberrant GnRH cell migratory pathway in the wild-type and the mutant mouse, respectively. In both cases, migrating GnRH cells appear to follow peripherin-immunoreactive axonal fibers (arrows). Abbreviations: cx, cortex; nm, frontonasal mesenchyme; ob, olfactory bulb; vfb, ventral forebrain. Scale bar: 50 µm (20 µm in insets). (TIF) [file pgen.1002896.s002.tif]

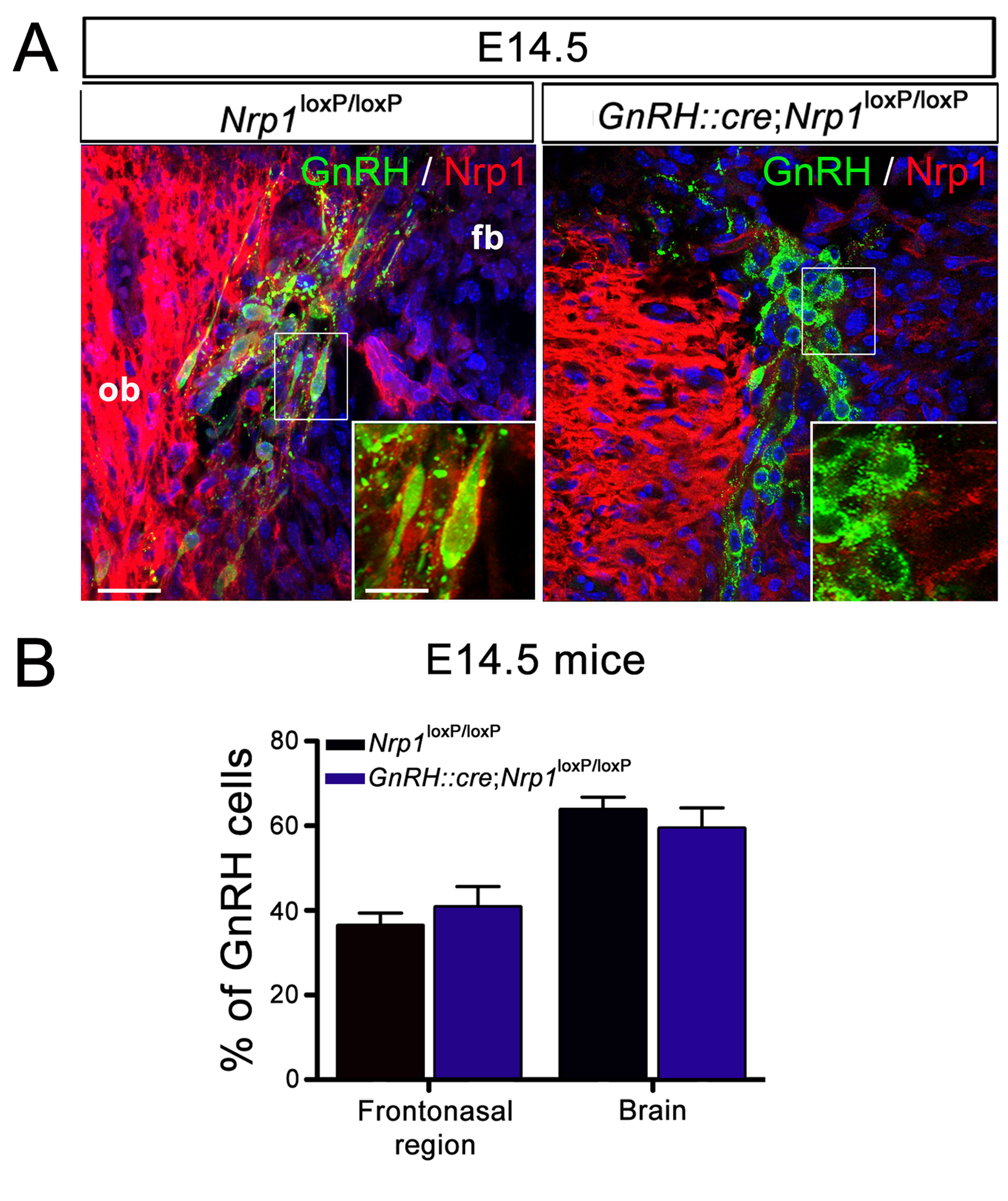

Supplement: Figure S3 — GnRH cell migration is not affected in GnRH::cre; Nrp1 loxP/loxP conditional knockout mice that lack Nrp1 only in GnRH cells. (A) Immunohistofluorescence analysis of the frontonasal region (sagittal sections, single plane confocal microscopy images) in Nrp1 loxP/loxP and GnRH::cre; Nrp1 loxP/loxP mice at E14.5, with anti-GnRH (green) and anti-Nrp1 (red) antibodies. As expected, Nrp1 immunoreactivity of the GnRH cells (yellow) is detected in the Nrp1 loxP/loxP mouse, but not in the GnRH::cre; Nrp1 loxP/loxP mouse. Abbreviations: ob, olfactory bulb; fb, forebrain. Scale bar: 50 µm (20 µm in insets). (B) Nrp1 loxP/loxP and GnRH::cre; Nrp1 loxP/loxP mice display similar distributions of GnRH cells between the nose and the brain at E14.5 (Kruskal-Wallis test, p>0.05). (TIF) [file pgen.1002896.s003.tif]
