# Supplementary material for: SEMA3A, a Gene Involved in Axonal Pathfinding, Is Mutated in Patients with Kallmann Syndrome
Source: PLoS Genet. 2012 Aug 23;8(8):e1002896. doi: 10.1371/journal.pgen.1002896 (PMC3426548; doi:10.1371/journal.pgen.1002896)
Supplement: Table S1 — SEMA3A sequencing primers. (DOCX) [file pgen.1002896.s004.docx]

**Table S1: Set of primers for Sanger sequencing of *SEMA3A* exons**

SEMA3A-1A: GAAACTGACCTAAATCACCTG

SEMA3A-1B: GTTGGGAGGGAGTTCAAGG

SEMA3A-2A: ACAGCAGTCAATATAGTCAGG

SEMA3A-2B: TATCTATAACTTGAGATGCTTC

SEMA3A-3A: CAGTTGCCCCAATGTCATCT

SEMA3A-3B: GAATGAAGGATACTCAACCTG

SEMA3A-4A: ATCTGTTCCAGCATGCCTAG

SEMA3A-4B: TGAATAGAAAGGGGTCATGGA

SEMA3A-5A: TCTCTGATTAACTGATGTGTTG

SEMA3A-5B: AAAGAACATACAACCTGTTTGT

SEMA3A-6A: ATGGTCATAACATGAAACTTGC

SEMA3A-6B: TCAAGTCATATTGCATGTACTG

SEMA3A-7A: AATGGACTGTTCAGAATGGTAT

SEMA3A-7B: TTGTATATGCACACAGGTAGAA

SEMA3A-8A: TTGAGGGAACGATTCGACC

SEMA3A-8B: TCTATACATAAACACTAGCTTC

SEMA3A-9A: AACTGCGTAAGAAGCTAGTGT

SEMA3A-9B: TATGAGTACTTGGATAGCACC

SEMA3A-10A: CTCAGTATCAATATTTCTCTTAC

SEMA3A-10B: TATCTGTCTGTAGCTGCATTG

SEMA3A-11A: AGAACCATTGAGGCCATGTG

SEMA3A-11B: CATCCCAACCCCTGAGATG

SEMA3A-12A: GGAAGACCGATATCAAAGGTT

SEMA3A-12B: GGAAAGACGTACAACTGAACT

SEMA3A-13A: ACTAATGGCAGTGCCTTGAG

SEMA3A-13B: CTAATCTACTAGCTTATTGTAAG

SEMA3A-14A: GAACAAGAGATTTAAAAGACAAG

SEMA3A-14B: ACTTGGAATCAGATAGGATAAC

SEMA3A-15A: TTGGAGACTGCTCTTACAGG

SEMA3A-15B: CTTTAAGTATTCTGAGAGATGC

SEMA3A-16A: TTGGCAATAACTTGTCTCCTG

SEMA3A-16B: AGGATAAGCATTCTCAGTGCT

SEMA3A-17A: ACACGGAGTTTCAGAGCTTC

SEMA3A-17B: GGAACTCAGCTGAATTTCCC
