# Supplementary material for: SEMA3A, a Gene Involved in Axonal Pathfinding, Is Mutated in Patients with Kallmann Syndrome
Source: PLoS Genet. 2012 Aug 23;8(8):e1002896. doi: 10.1371/journal.pgen.1002896 (PMC3426548; doi:10.1371/journal.pgen.1002896)
Supplement: Table S2 — NRP1 sequencing primers. (DOCX) [file pgen.1002896.s005.docx]

**Table S2: Set of primers for Sanger sequencing of *NRP1* exons**

NRP1-1A: GAGGATTGTACAGCTCTAGG

NRP1-1B: TCGGTTGTTCCCGGCTGAT

NRP1-2A: GCTAGATTACCTAACAGGTTG

NRP1-2B: AGACAGGCGTGACCACTAG

NRP1-3A: AAGGGCATTTCTCACCAACC

NRP1-3B: GCCACCACACTCGGCCTA

NRP1-4A: ATGTTCTGTCTTTACCCAGGT

NRP1-4B: GATTCATGTATCATGAGACTTG

NRP1-5A: TGCTAATTCTTGCATCTGCTG

NRP1-5B: CATGTGGCCGCAGGTGTG

NRP1-6A: TCCAAGTATCAGTGCTATATTCC

NRP1-6B: TCAACAACCTCTCTAGATGGT

NRP1-7A: CCTGTTGATCCCAGGTGGA

NRP1-7B: GGCCAGACAGAAAGCTACC

NRP1-8A: ATCTCATCCTTGATCGACAAC

NRP1-8B: CATTTACAAACTTATTTACCCTG

NRP1-9A: TGAGGGAGGAAAGATTTACTG

NRP1-9B: TCCTCTAATGTCATGGCTGG

NRP1-10A: ACAAAGTGGTAGAATGAAGCC

NRP1-10B: TTTGCAAAGGCACCATCAGG

NRP1-11A: TCCAACAGCCAGAGGCTTG

NRP1-11B: GTTAGCTGGTCCCAACTAAG

NRP1-12A: CTCTGATAGACATTTGTAAGCA

NRP1-12B: CCAGATGCTGTGTGGCATC

NRP1-13A: TTCCATGAGTGAACCAAGGG

NRP1-13B: ATTCACAGACATTAGAAACCCT

NRP1-14A: GTGGTAATCCTGGCCACTC

NRP1-14B: TCAGCATTCCTGCAGCCAC

NRP1-15A: TAGCTGGAGCTGAACAAGCA

NRP1-15B: AGACCATCATATTGGCAAGAG

NRP1-16A: ATGTACCTAAAGGTTTGTGTAG

NRP1-16B: ACCAGTGTATTAGAGTTCCAC

NRP1-17A: GTGCTAAGCAGTCTGTCAGA

NRP1-17B: AAGATCAACAGCTCCCCCAGC
